# The interaction of intestinal microbiota and diabetes mellitus: A database-based quantitative analysis and visualization

**DOI:** 10.12669/pjms.42.8.13682

**Published:** 2026-08

**Authors:** Qian Wang, Liwen Zhan, Ying Wen, Chenyang Wang

**Affiliations:** 1 Qian Wang Department of Clinical Laboratory, The Hospital of 82nd Group Army PLA, Baoding 071000, Hebei, China; 2 Liwen Zhan Department of Clinical Laboratory, Hebei Provincial Mental Health Center, Baoding 071000, Hebei, China. The Sixth Clinical Medical College of Hebei University, Baoding 071000, Hebei, China; 3 Ying Wen Department of Ultrasound, The Hospital of 82nd Group Army PLA, Baoding 071000, Hebei, China; 4 Chenyang Wang Central Laboratory, Department of Cardiology, The Hospital of 82nd Group Army PLA, Baoding 071000, Hebei, China

**Keywords:** Diabetes Mellitus, Interaction, Intestinal Microbiota, Quantitative Analysis, Visualization

## Abstract

**Objective:**

To comprehensively characterize the composition and structure of intestinal microbiota in Type-II diabetes mellitus (T2DM) patients and identify microbial taxa associated with metabolic parameters, particularly body mass index (BMI), using integrated multi-database analysis and quantitative visualization approaches.

**Methodology:**

This retrospective study was conducted at The Hospital of the 82nd Group Army of the PLA from 2024 to 2025, using data from public repositories. Using multi omics sample analysis methods, quantitative analysis and visualization of the interactions between gut microbiota and diabetes mellitus were discovered and explained. The original data was obtained from the NCBI and GMrepo databases. The abundance of gut microbiota was analyzed and visualized in diabetic patients of various BMI indexes, sexes, and ages.

**Results:**

A comprehensive analysis of 2,457 bacterial species and 550 genera revealed Bacteroides as the most abundant genus (11.29%), followed by *Faecalibacterium* (4.70%). Bacteroides, particularly *B. fragilis*, correlated with BMI and was consistently abundant across ages. In diabetic patients, females exhibited higher microbial abundance than males, with Bacteroides representing 20.95% and 18.49%, respectively.

**Conclusion:**

This study offers a comprehensive view of the interplay between intestinal microbiota and diabetes mellitus in patients.

## INTRODUCTION

By 2030, diabetes is projected to affect 578 million people globally.^1^-^3^ It is categorized primarily into Type-I, resulting from autoimmune destruction of pancreatic β cells, and Type-II, characterized by insulin resistance and relative insulin deficiency.^4^,^5^ A minority of cases involve other forms, such as juvenile or latent autoimmune diabetes.^6^,^7^ Although adults represent most cases of both types, prevalence data in U.S. adults remain limited, partly due to historical challenges in classifying diabetes by type.^8^

Diabetics often develop complications such as kidney failure, retinopathy, neuropathy, cardiovascular disease, and limb amputations. Altered gut microbiota composition has been strongly associated with metabolic disorders, including diabetes,^9^,^10^ and several microbial mechanisms underlying T2DM onset and progression have been proposed.^11^ Recent studies indicate that intestinal microbiota is closely linked to insulin dysfunction. Advances in high-throughput sequencing and multi-omics have enhanced our understanding of diabetes and facilitated practical applications, prompting increased research into informative tools for clinicians and scientists.^12^ However, the complexity of microbiota data and high-throughput outputs pose challenges for obtaining clear and actionable insights.

Thus, this study quantitatively analyzes and visualizes microbial abundance and other key data from multiple databases to provide intuitive, concise, and insightful information for the field.

## METHODOLOGY

This was a retrospective database analysis. The study conducted at The Hospital of the 82nd Group Army of the PLA from 2024 to 2025, using data from public repositories, and utilized publicly available genomic and clinical data. Data on gut microbiota in Type-II diabetes were obtained from Gut Microbiota (GMrepo) and The National Center for Biotechnology Information (NCBI), including patient metadata and microbial sequence data (BioProject: PRJEB11419).^13^

### Ethical approval:

This study involved the analysis of de-identified, publicly available data. Therefore, it was granted an exemption from requiring ethical approval by the Institutional Review Board of The Hospital of the 82nd Group Army of the PLA (No: 2023022, dated March 15, 2023). All original data collection procedures in the source databases were conducted in accordance with the ethical guidelines of their respective institutions and informed consent protocols. No additional informed consent was required for this secondary analysis of de-identified data.

### Inclusion criteria:

- Within the specified Bio Project (PRJEB11419) that were explicitly annotated as originating from patients diagnosed with T2DM.
- Containing both 16S rRNA gene sequencing data and complete metadata (including age, sex and BMI).

### Exclusion criteria:

- Samples from healthy controls, patients with other disease types.
- With incomplete metadata.

### Data preprocessing and Bioinformatics workflow:

Sequence Read Archive file were converted to FASTQ format using SRA Toolkit (v2.9.6). Paired-end reads were separated using SeqKit (v0.16.0) and merged using USEARCH (v11.0.667). Merged FASTQ files from multiple samples were combined for batch processing.

Quality filtering and primer trimming (left 19 bp, right 21 bp) were performed using VSEARCH (v2.13.6) with a maximum expected error rate of 0.01. Unique sequences were dereplicated (minimum abundance eight). Sequences were clustered at 97% identity. For ASV inference, UNOISE (minimum size 10) was applied. Chimeras were removed using VSEARCH. OTU tables are generated by comparing filtered sequences with the SILVA database (V123, similarity threshold of 97%). Relative abundance was normalized to 100,000 reads per sample. Taxonomic classification was performed with the SILVA database using SINTAX with a confidence cutoff of 0.1.

### Statistical analysis:

Statistical analyses were conducted using Free Statistics software.^13^ Data analysis and visualization were performed using R (v4.2.2) and Origin Pro 2021. The pheatmap (v1.0.12), corrplot (v0.92), and Venn Diagram (v1.10) packages were applied for species abundance, correlation, and Venn analyses, respectively. The significance threshold (p < 0.05) and all software versions (Free Statistics v1.1.4, R v4.2.2, Origin Pro 2021) are now specified.

## RESULTS

The sample contains 25 phyla, with *Proteobacteria*, *Firmicutes*, *Bacteroidetes*, *Verrucomicrosbia*, *Actinobacteria*, Teneris, *Cyanobacteria*, *Fusobacteria*, Synergites, and *Euryarchaeota* predominating. There are 36.4%, 33.2%, 26.9%, 1.53%, 1.23%, 0.46%, 0.10%, 0.05%, 0.04%, and 0.03% of each phylum.

At the genus level, there are 494 genera classified, of which 187 belong to the phylum Firmicutes, 178 to the phylum Proteobacteria, 59 to the phylum Actinobacteria, 44 to the phylum Bacteroidetes, seven to the phylum *Acidobacteria*, four to the phylum *Verrucomicrobia*, and three to the phylum Fusobacteria. Firmicutes have the most diverse flora, followed by Proteobacteria, Actinobacteria, and Bacteroidetes.

Firmicutes, *Proteobacteria*, *Bacteroides*, *Verrucomicrosbia*, and Actinobacteria are the top 30 genera, with 15, 9, 4, 1, and 1, respectively. Table-I shows the relative abundance of the top 30 dominant genera flora.

**Table-I:** Genera ranking of the first 30 intestinal flora in diabetes mellitus.

Genus name	Relative abundance(%)
Bacteroides	19.75
Escherichia-Shigella	14.87
Faecalibacterium	5.80
Klebsiella	4.46
Salmonella	3.71
Hafnia	3.06
Subdoligranulum	2.12
Alistipes	2.06
Acinetobacter	1.65
Pseudobutyrivibrio	1.63
Ruminiclostridium	1.55
Parabacteroides	1.55
Akkermansia	1.52
Ruminococcus	1.50
Prevotella	1.44
Blautia	1.39
Enterobacter	1.33
Rummeliibacillus	1.32
Citrobacter	1.22
Pseudomonas	1.11
Roseburia	1.06
Lachnoclostridium	1.06
Ruminococcaceae UCG-002	1.06
Christensenellaceae R-7 group	0.88
Ruminococcaceae UCG-014	0.85
Bifidobacterium	0.76
Stenotrophomonas	0.76
Lachnospiraceae NK4A136 group	0.72
Coprococcus	0.64
Dialister	0.58

**Fig.1:**
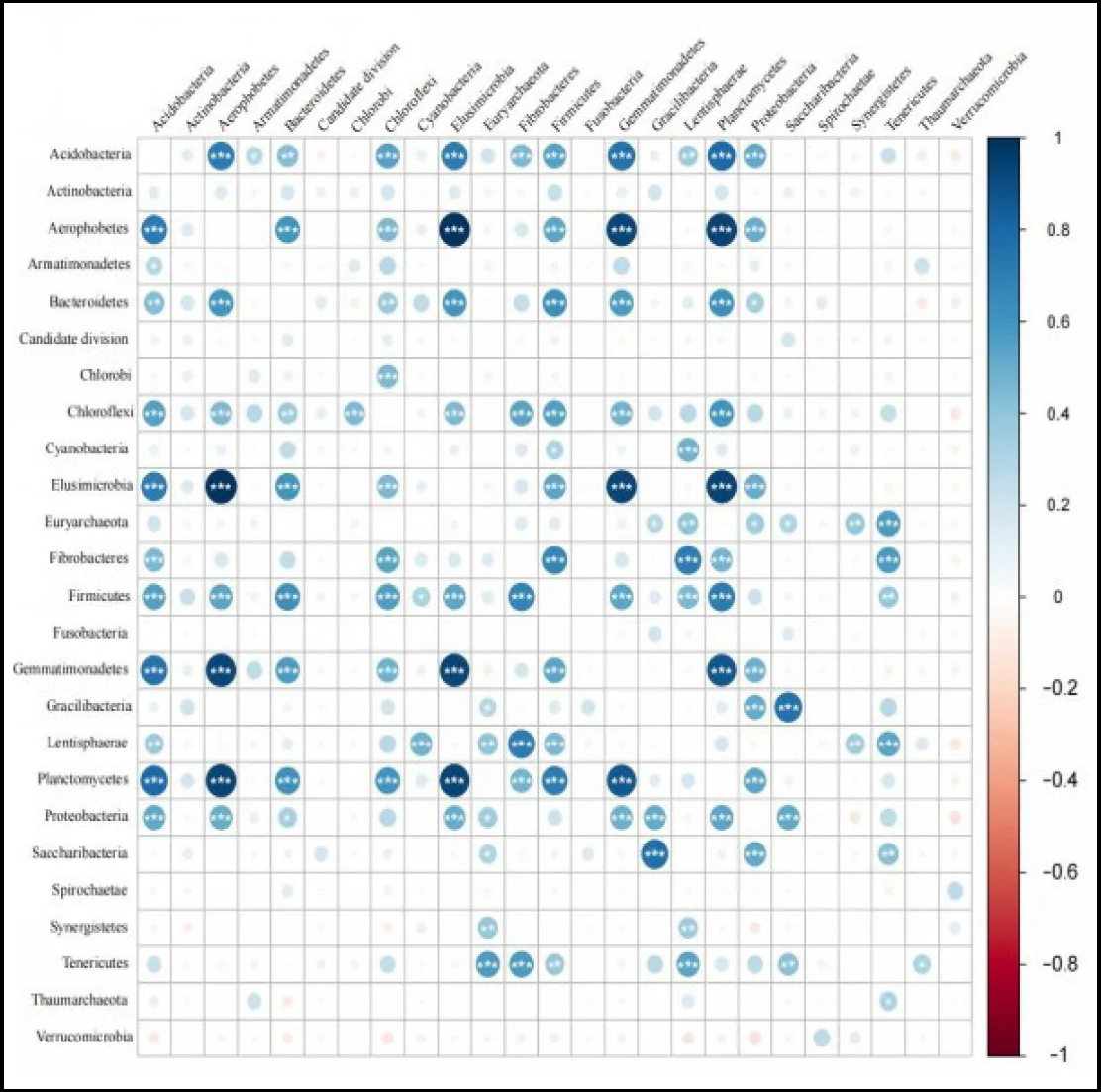
Relativity analysis of flora microbiota based on multi-samples.

In addition, a correlation analysis of the top 25 phyla of intestinal microbiota was performed (Fig.1). *Aerophobetes* was found to be related to *Planctmycetes*. *Elusimicrobia* was positively correlated with *Gemmatimonadetes*. Only a few of the phyla have a negative correlation overall. The correlation’s significance is not obvious.

**Fig.2:**
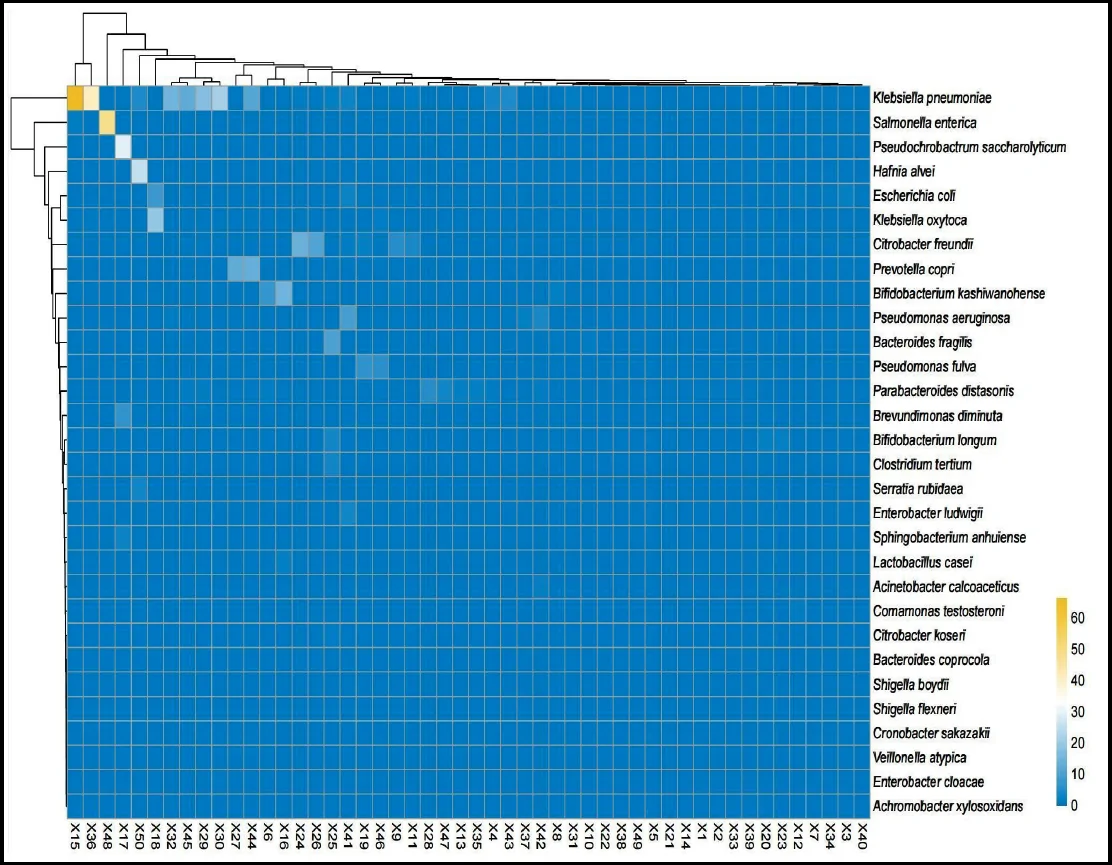
Visualization of Relative Abundance of Intestinal Flora in Different Samples of diabetes mellitus.

We investigated the NCBI BioProject PRJEB11419 project. The relative abundance of the top 30 species was visually displayed (Fig.2). Furthermore, the correlation analysis revealed that much of the flora had a strong positive correlation (correlation > 0.5), with only a few flora having a strong negative correlation. This suggests that most intestinal microflora abundance changes are consistent, indicating that the dominant microflora may be interdependent. There are strong positive correlation between Bacteroides fragilis and Clostridium tertium, *Sphingobacterium* anhuiense and Pseudochrobactrum saccharolyticum, Hafnia alvei and Serratia rubidaea.

**Fig.3:**
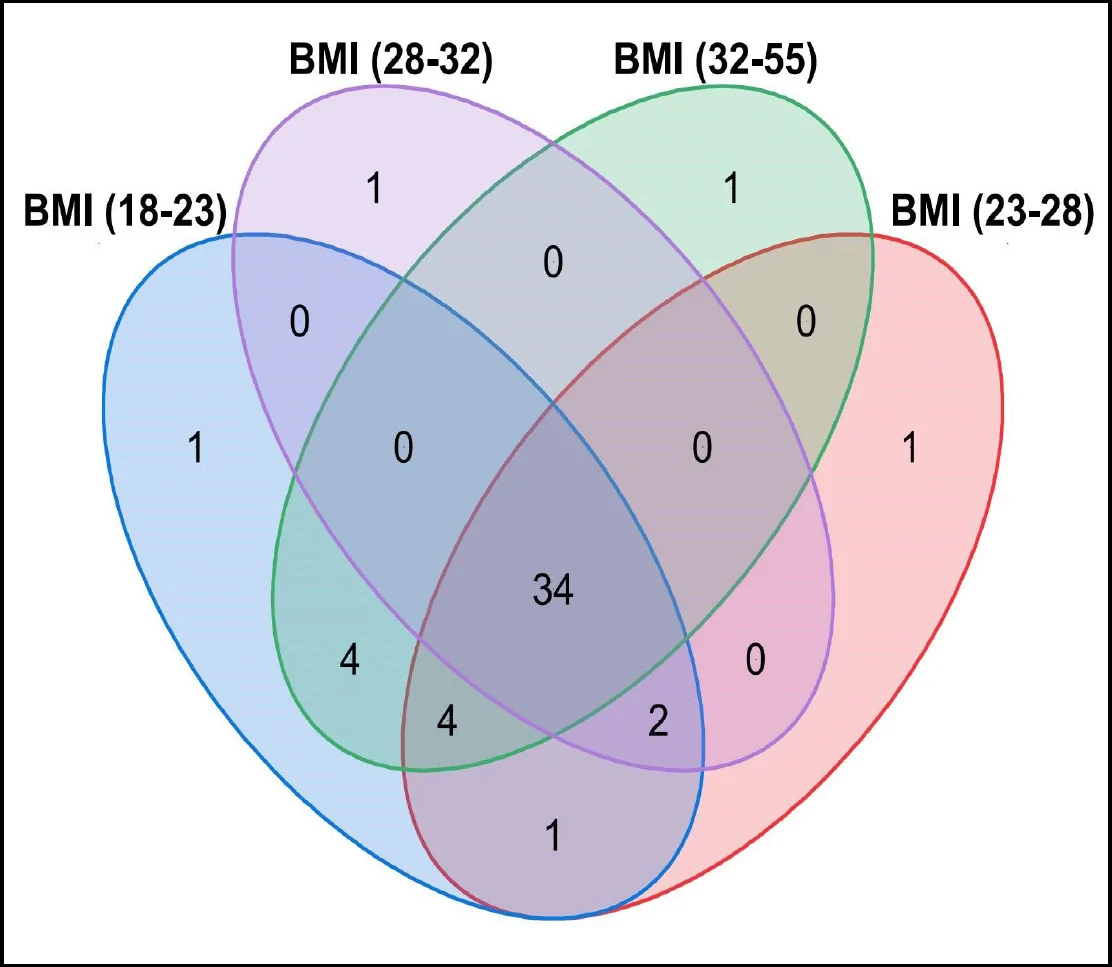
Intersection of top 30 intestinal microbiota in abundance based on BMI categories.

BMI is classified into five different categories based on China’s reference values: underweight (BMI < 18 kg/m²), normal weight (18 ≤ BMI < 24 kg/m²), overweight/pre-obesity (24 ≤ BMI < 28 kg/m²), obesity class I (28 ≤ BMI < 32 kg/m²), and obesity class II (BMI ≥ 32 kg/m²). The average abundance of gut microbiome in the five groups was calculated, and the first 50 were chosen to visualize by Wayne analysis (Fig.3).

**Fig.4:**
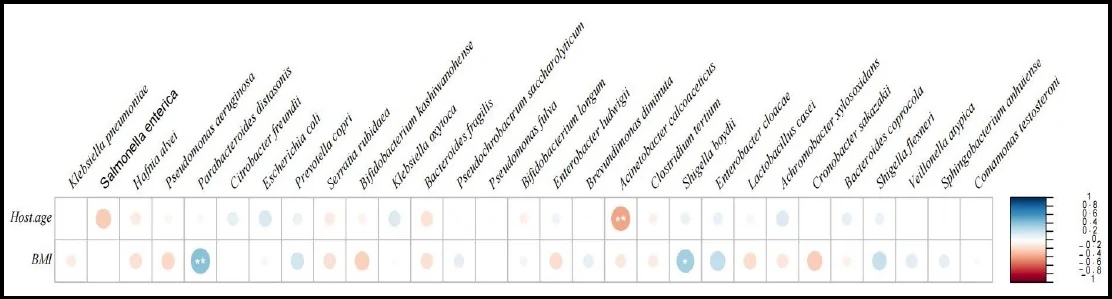
Correlation analysis between the top 30 species and BMI.

*Pseudochrobactrum saccharolyticum* is abundant in obesity class II (BMI ≥ 32 kg/m²), whereas *Bifidobacterium* kashiwanohense is abundant in underweight (BMI < 18 kg/m²). *Bifidobacterium kashiwanohense* was not detected in overweight/pre-obesity (24 ≤ BMI < 28 kg/m²) or obesity class I (28 ≤ BMI < 32 kg/m²) groups. The findings revealed that the relative abundance of Paraacteroides distasonis and Shigella boydii increased along with BMI; however, many species had a negative correlation with BMI that was not statistically significant (Fig.4). The correlation analysis between various species relative abundance of Bacteroides and BMI also showed that the relative abundance of Bacteroides fragilis have a negative correlation with BMI. Further use the Free Statistics analysis platform software to analyze the correlation between Top 30 species and BMI groups. The results show that BMI and Bacteroides Fragilis has a strong negative correlation, with p = 0.031. That is, with the increase of BMI, the flora abundance of Bacteroides fragilis in the intestinal flora of patients with Type-II diabetes decreases accordingly.

## DISCUSSION

This study adds to the literature by providing a structured, visual, and quantitative framework for analyzing high-throughput microbiota data in T2DM, integrating multi-database sources to improve reproducibility and insight extraction. We demonstrate the utility of combining correlation analysis, abundance profiling, and BMI stratification to identify clinically relevant microbial patterns. Type-II diabetes predominates, accounting for 91% of cases versus 6% for Type-I.^14^ Prevalence among adults aged 20–79 has plateaued.^15^ Altered gut microbiota is strongly associated with diabetes, involving multiple proposed molecular pathways in T2DM.^11^

The gut microbiota is predominantly composed of six phyla: *Firmicutes*, *Bacteroidetes*, *Actinobacteria*, *Proteobacteria*, *Fusobacteria*, and *Verrucomicrobia*, with *Firmicutes* and *Bacteroidetes* typically constituting over 90% of the community.^16^ In contrast, this study observed these two phyla represented only 60%, while Proteobacteria accounted for 36.4%. This discrepancy may stem from regional dietary patterns, genetic background, or methodological differences in sequencing and bioinformatics. Notably, an elevated abundance of Proteobacteria is often considered a microbial signature of dysbiosis and elevated metabolic disease risk,^17^ aligning with its prominence in our T2DM samples.

Obesity, a major Type-II diabetes risk factor, promotes low-grade inflammation and insulin resistance through pro-inflammatory cytokines that disrupt insulin signaling and intestinal barrier function. Although Gram-negative bacterial LPS contributes to inflammation, Bacteroidetes produce immunoinhibitory LPS variants.^18^ Bacteroides fragilis exhibits virulence traits including tissue adhesion and capsule protection, and its abundance is influenced by diet-especially high fat and protein intake.^19^ Enterotoxigenic strains (ETBF) are associated with metabolic and inflammatory diseases, though some may aid immune development.

The finding was the significant negative correlation between *Bacteroides fragilis* abundance and BMI (p=0.031), which contrasts with several international reports linking *B. fragilis* (especially enterotoxigenic strains) to obesity and inflammation. This divergence may be attributable to ethnic differences, strain-specific variations, or interactions with host diet-particularly typical carbohydrate and fat intake in the study population.^18^ Our multi-project integrative analysis further uncovered specific inter-species correlations, such as between *B. fragilis* and *Clostridium tertium*, suggesting potentially unique ecological interactions in T2DM patients that have not been widely reported.

### Strengths of this study:

It include the use of publicly available datasets with detailed metadata, rigorous statistical visualization, and a focus on contextualizing results within a clinically relevant framework (BMI categories).This manuscript conform the Enhancing the Quality and Transparency Of health Research (EQUATOR) network guidelines.

### Novelty of the study:

Unlike previous single-center or single-database studies, this research integrates data from multiple public repositories (NCBI and GMrepo), enabling a larger sample size and greater generalizability. The novel finding of a significant negative correlation between *Bacteroides* fragilis and BMI in T2DM patients contrasts with previous international reports, suggesting important ethnic and population-specific variations in microbiota-metabolic associations. Additionally, this study employs a systematic, quantitative visualization approach that translates complex omics data into clinically actionable insights, a methodological innovation that has not been extensively reported in previous T2DM microbiota studies.

### Limitations:

Despite integrating multiple databases, the sample size remains modest and the retrospective, observational design cannot establish causal relationships. The exclusive use of 16S rRNA sequencing restricts functional insights, and potential batch effects from heterogeneous sequencing platforms and collection procedures may exist. Key confounders such as antibiotic use, diet, and medication history were not uniformly available for adjustment. Future research should validate these findings in larger, prospective cohorts with standardized metadata and employ metagenomic, metabolomic, or animal models to explore causality, particularly the role of *B. fragilis* in glucose metabolism and its therapeutic potential.

## CONCLUSIONS

This study offers a novel approach and wide-ranging viewpoints for assessing the relationship between diabetes mellitus and intestinal microbiota in patients. These findings offer a valuable reference for future clinical and mechanistic studies, supporting the development of microbiota-based diagnostics or interventions for diabetes management.

## Authors’ Contributions:

**QW** and **CW:** Conceived and designed the study, drafted the manuscript, and are responsible and accountable for the accuracy and integrity of the work.

**LZ:** Literature search, Analyzed data.

**YW:** Critical Review, Interpreted results and prepared figures.

All authors have read and approved the final manuscript.

## References

[1] Chen X, Zhang L, Chen W (2025). Global, regional, and national burdens of Type-I and Type-II diabetes mellitus in adolescents from 1990 to 2021, with forecasts to 2030: a systematic analysis of the global burden of disease study 2021. BMC Med.

[2] Liu J, Liu M, Chai Z, Li C, Wang Y, Shen M (2023). Projected rapid growth in diabetes disease burden and economic burden in China: a spatio-temporal study from 2020 to 2030. Lancet Reg Health West Pac.

[3] Xu Y, Lu J, Li M, Wang T, Wang K, Cao Q (2024). Diabetes in China part 2: prevention, challenges, and progress. Lancet Public Health.

[4] Hu J, Jiang G, Qin J, Luo S, Fan B, Xie Z (2025). A Type-I diabetes genetic risk score discriminates between Type-I diabetes and Type-II diabetes in a Chinese population. Diabetologia.

[5] Albanese I, Rendon L, Nakhla M, Rahme E, Dasgupta K (2025). Gestational diabetes and parental Type-II diabetes as risk indicators for Type-I diabetes in offspring: a systematic review and meta-analysis. EClinical Medicine.

[6] Cheng L, Zhang D, Chen B (2015). Declined plasma sfrp5 concentration in patients with Type-II diabetes and latent autoimmune diabetes in adults. Pak J Med Sci.

[7] Luckett AM, Hawkes G, Green HD, De Franco E, Hagopian WA, Roep BO (2025). Type-I Diabetes Genetic Risk Contributes to Phenotypic Presentation in Monogenic Autoimmune Diabetes. Diabetes.

[8] Accili D, Deng Z, Liu Q (2025). Insulin resistance in Type-II diabetes mellitus. Nat Rev Endocrinol.

[9] Li L, Tang L (2025). Gut Microbiota in Exercise-Regulated Development, Progression, and Management of Type-II Diabetes Mellitus: A Review of the Role and Mechanisms. Med Sci Monit.

[10] Keivanlou MH, Amini-Salehi E, Sattari N, Hashemi M, Saberian P, Prabhu SV (2024). Gut microbiota interventions in Type-II diabetes mellitus: An umbrella review of glycemic indices. Diabetes Metab Syndr.

[11] Gurung M, Li Z, You H, Rodrigues R, Jump DB, Morgun A (2020). Role of gut microbiota in Type-II diabetes pathophysiology. EBioMedicine.

[12] Fan Y, Pedersen O (2021). Gut microbiota in human metabolic health and disease. Nat Rev Microbiol.

[13] McDonald D, Hyde E, Debelius JW, Morton JT, Gonzalez A, Ackermann G (2018). American Gut: an Open Platform for Citizen Science Microbiome Research. mSystems.

[14] Bullard KM, Cowie CC, Lessem SE, Saydah SH, Menke A, Geiss LS (2018). Prevalence of Diagnosed Diabetes in Adults by Diabetes Type - United States, 2016. MMWR Morb Mortal Wkly Rep.

[15] Fu P, Wen J, Duan X, Hu X, Chen F, Yuan P (2024). Association between adult food insecurity and mortality among adults aged 20-79 years with diabetes: A population-based retrospective cohort study. Diabet Med.

[16] Rinninella E, Raoul P, Cintoni M, Franceschi F, Miggiano GAD, Gasbarrini A (2019). What is the Healthy Gut Microbiota Composition? A Changing Ecosystem across Age, Environment, Diet, and Diseases. Microorganisms.

[17] Banta AB, Hall AN, Myers KS, Ward RD, Davis BC, Cuellar RA (2025). Targeted, Genome-scale Overexpression in Proteobacteria. Preprint. bioRxiv.

[18] d'Hennezel E, Abubucker S, Murphy LO, Cullen TW (2017). Total Lipopolysaccharide from the Human Gut Microbiome Silences Toll-Like Receptor Signaling. mSystems.

[19] Sun F, Zhang Q, Zhao J, Zhang H, Zhai Q, Chen W (2019). A potential species of next-generation probiotics? The dark and light sides of Bacteroides fragilis in health. Food Res Int.

